# Aortic aneurysm: pathophysiology and therapeutic options

**DOI:** 10.1002/mco2.703

**Published:** 2024-09-07

**Authors:** Guang Yang, Abbas Khan, Wei Liang, Zibo Xiong, Johannes Stegbauer

**Affiliations:** ^1^ Division of Renal Medicine Peking University Shenzhen Hospital Shenzhen China; ^2^ Shenzhen Institute of Translational Medicine Shenzhen Second People's Hospital The First Affiliated Hospital of Shenzhen University Shenzhen China; ^3^ Department of Life Sciences Yuncheng University Yuncheng China; ^4^ Shenzhen Clinical Research Center for Urology and Nephrology Shenzhen China; ^5^ Department of Nutrition and Health Promotion University of Home Economics Lahore Pakistan Lahore Pakistan; ^6^ Department of Nephrology Medical Faculty University Hospital Düsseldorf Heinrich Heine University Düsseldorf Düsseldorf Germany

**Keywords:** aortic aneurysm, aortic lesion, inflammation, oxidative stress, renin–angiotensin system

## Abstract

Aortic aneurysm (AA) is an aortic disease with a high mortality rate, and other than surgery no effective preventive or therapeutic treatment have been developed. The renin–angiotensin system (RAS) is an important endocrine system that regulates vascular health. The ACE2/Ang‐(1–7)/MasR axis can antagonize the adverse effects of the activation of the ACE/Ang II/AT1R axis on vascular dysfunction, atherosclerosis, and the development of aneurysms, thus providing an important therapeutic target for the prevention and treatment of AA. However, products targeting the Ang‐(1–7)/MasR pathway still lack clinical validation. This review will outline the epidemiology of AA, including thoracic, abdominal, and thoracoabdominal AA, as well as current diagnostic and treatment strategies. Due to the highest incidence and most extensive research on abdominal AA (AAA), we will focus on AAA to explain the role of the RAS in its development, the protective function of Ang‐(1–7)/MasR, and the mechanisms involved. We will also describe the roles of agonists and antagonists, suggest improvements in engineering and drug delivery, and provide evidence for Ang‐(1–7)/MasR's clinical potential, discussing risks and solutions for clinical use. This study will enhance our understanding of AA and offer new possibilities and promising targets for therapeutic intervention.

## Graphical abstract

Activation of Ang‐(1–7)/MasR axis protects vascular health and antagonizes the adverse effects of Ang II/ATIR activation on the development of vascular dysfunction, atherosclerosis, and aortic aneurysms (AAs). The discovery of the Ang‐(1–7)/MasR pathway has provided an important therapeutic target for the prevention and treatment of AA. This review will outline the epidemiology of AA and current diagnostic and treatment strategies. Due to the highest incidence and most extensive research on abdominal AA, we will focus on AAA to explain the role of the RAS in its development, the protective function of Ang‐(1–7)/MasR, and the mechanisms involved. We will also describe the roles of agonists and antagonists, suggest improvements in engineering and drug delivery, and provide evidence for Ang‐(1–7)/MasR's clinical potential, discussing risks and solutions for clinical use.

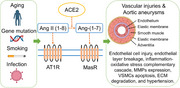

## 1 INTRODUCTION

Aortic aneurysms (AAs), including thoracic AA (TAA) and abdominal AA (AAA), are aneurysmal dilations of the aorta, usually recognized as aneurysms when the diameter increases by 50% or more. The mortality rate following rupture is as high as 65−85%.^1^, ^2^ Older populations are more susceptible to AA, with the incidence rising significantly above 65 years of age.^2^, ^3^, ^4^ The incidence rate is higher in males, but the risk of death is higher in females.^5^, ^6^, ^7^ Additionally, high‐risk causative factors include smoking, hypertension, atherosclerosis, coronary artery disease, diabetes, genetics, infection, and trauma.^5^, ^6^, ^8^, ^9^, ^10^, ^11^, ^12^ AA is usually considered a permanent and irreversible aortic injury. Currently, the best way to prevent and treat the development of AA is through screening and surgical procedure.^6^, ^13^, ^14^, ^15^ Early diagnosis and treatment can reduce the probability of AA development and improve survival outcomes.^16^, ^17^, ^18^ However, in practice, some studies have shown that surgical approaches are the only effective method for some AAAs’ patients.^19^ Moreover, patients with severe cardiopulmonary system disorders or coagulation disorders are not suitable for surgical procedures, forcing the search for more effective and universal prevention and treatment methods.

The renin–angiotensin system (RAS) is a crucial endocrine regulatory system that plays a role in regulating vascular tone and maintaining body fluid homeostasis. Numerous studies have demonstrated that although there are many reasons that trigger the formation of AA, many of them are related to dysregulation of the RAS. The entire pathway involves numerous peptides and receptors. This review focuses on two significant pathways, namely, the angiotensin converting enzyme (ACE)/angiotensin (Ang) II/Ang II type 1 receptor (ATIR) axis, and the ACE2/Ang‐(1–7)/Mas receptor (MasR) axis.^20^, ^21^ Upregulation of the ACE/Ang II/ATIR axis can elevate blood pressure, but excessive activation may induce aortic diseases such as hypertension, vascular dysfunction, atherosclerosis, and AA. Relevant inhibitors of this axis, such as ACE inhibitors captopril and enalapril, and Ang receptor blockers (ARBs) telmisartan, valsartan, and irbesartan, are commonly used as drugs to treat hypertension. In contrast, activation of the ACE2/Ang‐(1–7)/MasR axis antagonizes the effects of the former axis. The proven effects of this axis include reducing oxidative stress, suppressing inflammatory cell infiltration, inhibiting macrophages polarization toward M1, and mitigating vascular tissue injuries.^20^, ^21^, ^22^, ^23^, ^24^ At present, several agonists and antagonists targeting this axis have been discovered in preclinical studies, but no clinical drugs have been approved. Although numerous clinical drug trials have been conducted to treat AA, no drugs have been shown to reverse the formation of AA. Only a small number of drugs have been proven to alleviate the progression of AA.

Currently, the primary diagnostic method for AA is vascular morphology observation, while the only available prevention and treatment methods are surgical interventions. There are a few drugs available that target high‐risk factors before AA formation to prevent further exacerbation of vascular disease. These will be described in the coming sections. For nonsurgical indications of AA, patients need to take antithrombotic, hypolipidemic, and blood pressure‐lowering drugs for life, but these are used mainly to alleviate high‐risk factors for AA, without any proven ability to reverse AA or prevent its formation directly. Therefore, the development of AA drugs is relatively urgent, and the discovery of Ang‐(1–7)/MasR provides a potential opportunity. This study will initially provide an overview of the classification, preventative measures, and conventional clinical treatment strategies for AA. Given that AAA occurs more frequently than TAA and has been the subject of more extensive and illustrative research. We will concentrate more discussion on AAA. Additionally, we will delve into the associated risk factors and underlying mechanisms, with a particular focus on the role of the RAS in the pathophysiology of AAA. Following this, we will highlight the advancements in research surrounding Ang‐(1–7)/MasR related drugs, assessing their clinical potential and the risks associated with their use as pharmaceutical agents.

## 2 CLASSIFICATION OF AAs

### 2.1 Classification according to lesion location

AAs can be classified into various types, which differ in their characteristics and pathophysiological mechanisms. According to the anatomical location of the disease, they are primarily divided into AAA, TAA, and thoracoabdominal AA (TAAA).^18^, ^25^ AAA is the most common form of AA, typically occurring inferior to the renal arteries. This type is prone to rupture and carries a significant risk. TAA primarily affects the thoracic aorta and can be further categorized as ascending AA, aortic arch aneurysm, and descending AA. TAAs are also at risk of complications such as compression or rupture, with a similarly high‐risk profile. In comparison with these two types, the TAAA, which involves both the thoracic and abdominal aortic segments, is considered the most challenging to manage surgically.

### 2.2 Classification according to size

AAs can also be categorized based on their size. Generally, an aneurysm with a diameter less than 3 cm is classified as small, 3−5.5 cm as medium, 5.5 cm or greater as large, and 10 cm or greater as giant. However, this size‐based categorization exhibits regional variations depending on the aneurysm location. For AAA and TAA, a diameter exceeding 5.5 cm is considered large. In the case of TAAA, a diameter greater than 6 cm is defined as large. This size‐based classification criterion is primarily employed to assess the severity of aortic aneurysmal disease and to guide clinical management decisions. As a general principle, the larger the aneurysmal diameter, the higher the risk of rupture and associated complications.^26^

Typically, the renal artery diameter approximates 2 cm. Consequently, an AAA can be diagnosed when the aortic diameter exceeds 3 cm. If left untreated, the aneurysmal diameter will continue to progressively expand. The prevailing consensus has been that surgical repair is indicated for AAAs exceeding 5.5 cm in diameter.^27^ However, this conventional management approach has recently been challenged, with some experts advocating for surgical intervention when the AAA diameter surpasses 5 cm.^18^

### 2.3 Classification according to pathology

AAs can also be classified into three main categories: true AAs, pseudoaneurysms, and dissecting aneurysms.^28^, ^29^ True AAs are characterized by damage to the entire thickness of the aortic wall, resulting in an expanded aortic lumen that forms a sac‐like dilation. Examples of true AAs include those associated with atherosclerosis, syphilis, and hereditary conditions. In contrast, a pseudoaneurysm represents a partial disruption of the aortic wall, leading to blood flow outside of the aortic lumen and the formation of a sac‐like structure. Common examples of pseudoaneurysms include those caused by trauma or as a complication of previous surgical interventions.

Aortic dissection is a tear in the inner layer (intima) of the arterial wall, allowing blood to penetrate between the intima and the outer layer (adventitia) of the vessel, causing the wall to separate and dilate. This type of aneurysm occurs primarily in large arteries such as the aorta and carotid arteries. Consequently, the principal distinction between these three aneurysm classifications can be understood as a difference in the specific arterial wall layers that are compromised.

To assess the severity of the disease and to select treatment options, three types of dissecting aneurysm are classified clinically: the Stanford type, the DeBakey type, and the Svensson LG type.^29^ In type A, the intimal tear is located in the ascending aorta, the aortic arch, or the proximal descending aorta, with extension to the ascending aorta, the arch, and the descending and abdominal aorta. In type B, the intimal tear is often located in the isthmus of the aorta, with extension to the descending aorta or to the abdominal aorta only, but not to the ascending aorta. DeBakey classification divides dissecting aneurysms into three types. Type I is an intimal tear in the ascending aorta that affects the abdominal aorta. Type II is an intimal tear in the ascending aorta that extends primarily within the ascending aorta. Type III is an intimal tear in the isthmus of the aorta that may extend into the descending and abdominal aorta. The SvenssonLG classification system categorizes aortic dissections into five distinct grades. Grade I denotes an intimal tear between the true and false lumens of the dissection. Grade II refers to a rupture of the medial layer, frequently accompanied by intramural hemorrhage or hematoma formation. Grade III is characterized by the presence of fine dissections, but without any associated hematoma, eccentricity, or swelling at the site of the tear. Grade IV is classified as a plaque rupture or ulceration, with a hematoma under the adventitia. Grade V encompasses aortic dissections resulting from medical conditions or traumatic etiologies.

### 2.4 Uniqueness of AAA

While AAA, TAA, and TAAA may all formation within the aorta, and frequently in combination, they do not arise through precisely the same pathological mechanisms. The incidence of AAA in clinical practice is higher than that of TAA, and the occurrence of both types is believed to be associated with the RAS. In addition, the classical model of AAA in animals is based on the continuous perfusion of Ang II.^30^ Our research experience also suggests that Ang II predominately induces AAA, with occasional TAA or TAAA development. Moreover, Ang II causes injury to the entire aortic wall; consequently, Ang II‐induced AAA represents a true aneurysmal pathology. Given that the pathophysiology of these three distinct AA types cannot be generalized, the primary focus of the present study will be on AAA.

## 3 SCREENING, PREVENTION, AND TREATMENT OF AAS, AS WELL AS THE GENERAL PATHOPHYSIOLOGICAL PROCESSES INVOLVED IN THEIR FORMATION

**FIGURE 1:**
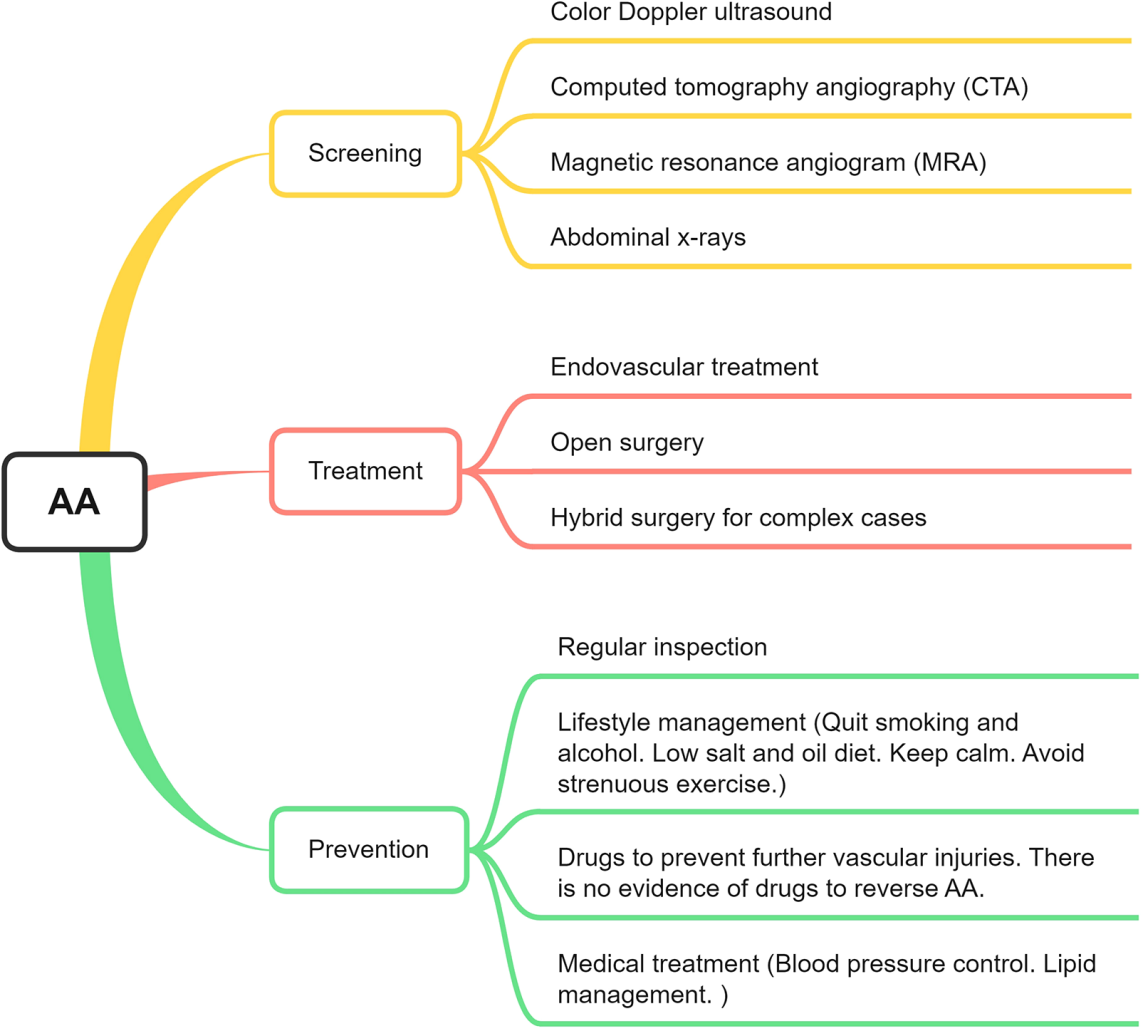
Screening, treatment, and prevention options for AA. The most common noninvasive diagnostic methods for AA are ultrasound, CT angiography (CTA), magnetic resonance angiography (MRA), and X‐ray. The most routine treatment methods are endovascular intervention and open surgery, and complex conditions may require a combination of both approaches. Prevention relies more on regular health check‐ups, lifestyle habit management, and medication to prevent further vascular injuries. However, no studies have proven that aneurysms can be reversed.

AA patients usually do not have any significant sign until aneurysms rupture. AAA patients with acute rupture present with sudden onset of severe pain in the lower back with signs of shock, and even death before hospital admission. Similarly, the main symptom of a TAA rupture is also severe pain, usually starting in the upper back and then possibly spreading to the lower back and abdomen. Some aneurysms are painful when they are about to rupture, so using pain to diagnose AA is a very dangerous practice. Hence, early screening and treatment are considered to be the key to preventing AA rupture. This section will present the existing screening, treatment, and prevention options for AA and discuss their pros and cons, as well as the necessity to develop new drugs (Figure 1).

### 3.1 Screening

It is now accepted that early screening and treatment of AAA is the best solution to prevent AAA rupture and can effectively reduce the public financial burden.^17^ Several vascular surgery societies recommend AAA screening with ultrasound in those above 65 years of age group. Early detection of asymptomatic AAA and surgery can significantly reduce emergency surgery and associated mortality from AAA.^19^ Conversely, some studies have also concluded that the lower prevalence in women does not allow them to significantly benefit from screening. Therefore, research studies recommended that screening should be done among different groups.^14^

In terms of screening, the following options are commonly used. First, color Doppler ultrasound is the preferred diagnostic method and it is highly accurate in detecting aneurysms, observe the diameter of the lumen, and displays information such as its shape and attached thrombus. Second, computed tomography angiography (CTA) is the most commonly used test for AA. Compared with ultrasonography, the complete picture of AA and its association with surrounding tissues such as renal artery, retroperitoneal and spinal structure, as well as complications such as retroperitoneal hematoma can be more clearly displayed. Third, CTA is preferred for the examination of aneurysms that have ruptured or dissected. Magnetic resonance angiogram (MRA) and angiography. It is primarily used as an evaluation tool in AA endovascular repair. In patients with renal insufficiency, MRA may be considered. Fourth, abdominal x‐rays can observe the typical oval shell‐shaped calcified shadow. The test results are not accurate because a small percentage of patients can have no results. Although these two methods can also be used as diagnostic tools for AA, but they are rarely used.

In addition, many scholars have proposed new screening and diagnostic methods, such as serum biomarkers.^15^, ^31^ But in fact, these methods can only be used as a reference, or as a pathological mechanism and therapeutic target. The final diagnosis of AA is mainly based on vascular morphological alterations.

### 3.2 Treatment

Once AA is diagnosed, it needs to be prevented from further deterioration and rupture by surgical approaches, mainly by endovascular treatment and open surgery.^16^ The first option is aortic stunting. A stent is implanted into the diseased aorta through a minimally invasive interventional approach, isolating the existing AA and allowing blood flow to pass through the stent. The second option is the traditional procedure of open AA resection with prosthetic vessel replacement. This involves the complete removal of the diseased aorta and the replacement of the diseased vessel with an artificial vessel reanastomosis. Endovascular surgery is a safer option than open surgery with less risk of injury, bleeding, re‐intervention, and complications, as well as good clinical success, survival and target vessel patency rates; and thus is more widely applicable.^32^, ^33^ However, it is noted that in the long term, there is little difference in mortality and reintervention after endovascular and open AAA repair.^34^ Recently, a third option, namely hybrid surgery, which is the combination of the aforementioned methods has also developed. This approach serves more complex cases.

### 3.3 Prevention

However, both procedures are associated with severe complications. A better option is prevention through noninvasive approaches. Unfortunately, there is no effective way to reverse AA. The most standard approach is to use regular follow‐up examinations, improve lifestyle habits, and taking medications to prevent vascular injuries, such as blood pressure‐lowering drugs, blood lipid‐lowering drugs, antithrombotic drugs, and anti‐inflammatory drugs. The details are described below.

There are two types of follow‐up examinations. When the aneurysm is less than 4 cm in diameter, an ultrasound examination is required once every 2−3 years. For those larger than 4 cm, patients need to be vigilant and have an ultrasound or CTA at least once a year.

In addition, better lifestyle management usually refers to abstaining from behaviors such as smoking and drinking that may increase vascular injuries. Eat a low‐salt and low‐oil diet to avoid elevated blood pressure and blood lipids. Avoid violent mood swings and strenuous exercise to reduce the risk of blood pressure and hormone fluctuations.

Furthermore, although there are no drugs specifically designed to prevent AA; however, medications that reduce vascular injuries can be used to prevent further vasculopathy. These medications include but are not limited to, antihypertensive drugs (β‐blockers), antiplatelet agents (aspirin, P2Y12 inhibitors, and cilostazol), statin lipid‐lowering drugs, inhibitors of immune/matrix metalloproteinases (MMPs) (cyclosporine), and PDE‐III inhibitors.^3^, ^35^, ^36^ Recent clinical studies have also shown that metformin can reduce the rate of AAA expansion, risk of rupture, and perioperative mortality.^37^, ^38^, ^39^, ^40^, ^41^ It has also been shown that hyperuricemia also accelerates the development of AAA; thus, it is recommended that controlling uric acid levels is also beneficial in mitigating AAA development.^42^ Nevertheless, it is important to note that these drugs only slow the progression of arterial disease, and there is no evidence that AA can be reversed.

These statements suggest that screening and surgery can only prevent AA rupture; and existing drugs may only mitigate the progression of AA‐related risk diseases. Hence, there is a lack of drugs that directly delay or reverse the development of AA. Therefore, we need to find a better and more effective therapeutic target.

### 3.4 General physiopathologic process of AA formation

**FIGURE 2:**
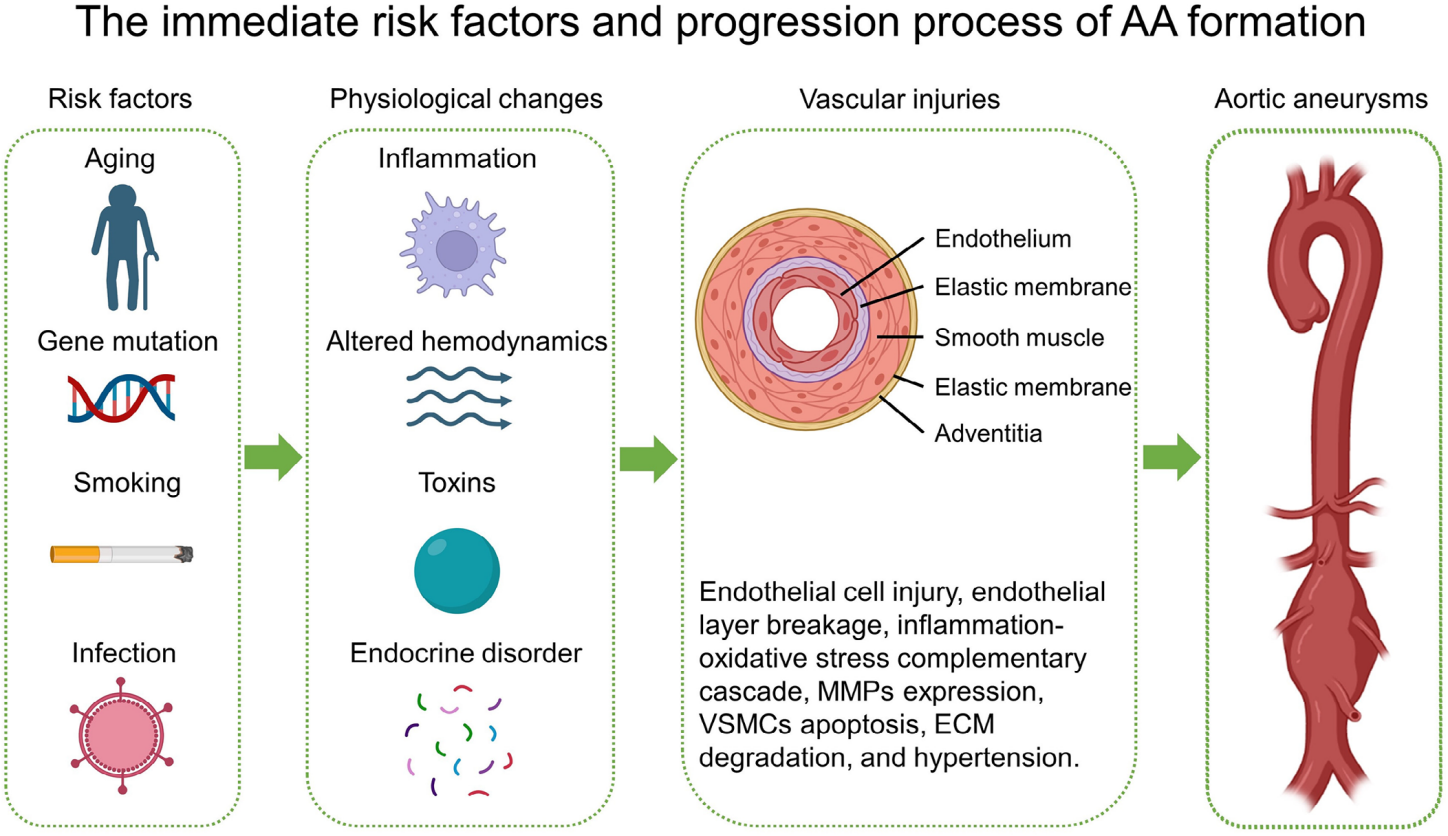
Schematic diagram of the causes of AA formation. Extrinsic and intrinsic risk factors can lead to physiological changes in blood vessels, including increased inflammation, changes in hemodynamics, accumulation of toxins, and endocrine disorders, causing vascular injuries. The continuous accumulation of these injuries can ultimately result in aortic pathologies, such as aneurysms. Created by Biorender software.

To effectively treat AA, it is crucial to understand the mechanisms underlying its formation. It is widely accepted that AA does not develop suddenly but progresses gradually (Figure 2). The process begins with endothelial injuries caused by toxins, altered hemodynamics, inflammation, and disturbances in regulatory factors.^43^, ^44^, ^45^ This process is also accompanied by lipid accumulation, activation of the inflammatory microenvironment, and the release of various factors that may damage the arterial wall, such as extracellular MMPs.^46^, ^47^ These proteases gradually degrade elastin and collagen fibers, which are the primary components responsible for maintaining the elasticity and dilation strength of the artery. The degradation and injury of both components cause a significant decrease in the mechanical strength of the abdominal aortic wall. In the presence of blood pressure, this leads to a limited expansion of the arterial wall into an aneurysm.

Furthermore, it has been suggested that aneurysms are a consequence of atherosclerosis.^48^ Atherosclerosis is frequently accompanied by inflammatory cell infiltration, which causes injury to the vessel wall, resulting in thinning of the vessel wall. The continuous impact of blood flow causes the wall to bulge, resulting in an aneurysm. Histological staining of AAAs showed marked endothelial damage and break, thickening of the media, and enlargement of the adventitia with disordered collagen arrangement, which was a typical phenomenon of cell death and collagen degradation caused by persistent inflammation.^22^, ^49^ These are the progressive processes of AAA pathology and there are many causes of these changes. Interestingly, large numbers of studies have shown that the RAS is involved in AAA formation induced by many kinds of factors. Details will be presented separately in the following sections.

## 4 ROLE OF RAS IN AAA FORMATION

### 4.1 RAS

**FIGURE 3:**
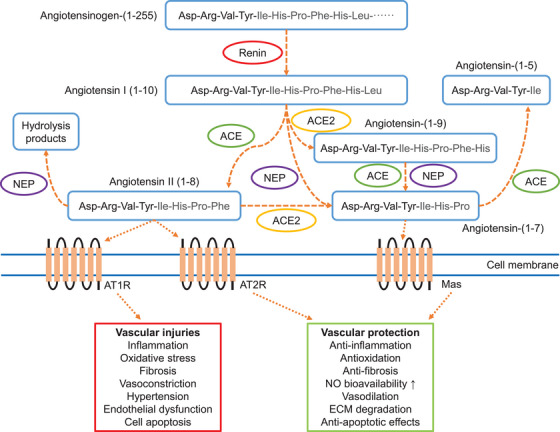
Illustration of the renin–angiotensin system, and its role in vascular pathophysiology. Ang‐(1–7) generation is dependent on ACE2 and NEP cleavage of upstream angiotensin proteins. ACE, angiotensin converting enzyme; ACE2, angiotensin converting enzyme 2; Mas, Ang‐(1−7) receptor; NEP, neprilysin; AT1R, Ang II type 1 receptor; AT2R, Ang II type 2 receptor; NO, nitric oxide; ECM, extracellular matrix. Picture adapted from Ref. 21.

Numerous studies have shown that AAA formation is closely associated with dysregulation of the RAS (Figure 3). Among these, upregulation or activation of the Ang II/AT1R axis is recognized as an important contributor to AAA formation and related arterial diseases. Subsequently, the Ang‐(1–7)/MasR axis was found to mitigate the unfavorable effects of the Ang II/AT1R axis in the development of vascular diseases. The discovery of these two axes is extremely important for AAA‐related investigations. This section will provide a brief overview of basic information on the RAS and focus on the role of Ang‐(1–7)/MasR in AAA formation due to Ang II/AT1R and other disease triggers. This will help us to understand the potential value of Ang‐(1–7)/MasR as a preventive and therapeutic drug for AAA and will provide evidences for its further clinical application.

The RAS is not only found in the circulatory system, but is also widely distributed throughout the body in many organs that function independently of each other.^50^, ^51^, ^52^ Local RAS may directly regulate cardiovascular activity by paracrine or autocrine ways.^53^, ^54^, ^55^ For example,^56^ overproduction of adipose‐specific angiotensinogen in mice increased plasma angiotensinogen levels by 30% and could potentially contribute to further hypertension and insulin resistance. In contrast, adipose‐specific angiotensinogen knockout reduced plasma angiotensinogen levels by 25%. This suggests an endocrine role of adipose RAS. Therefore, disorders of RAS regulation in a single organ/tissue may result in systemic changes in RAS levels, which in turn may increase the risk of cardiovascular diseases.

### 4.2 The classical RAS pathway ACE/Ang II/AT1R

The liver is the main source of angiotensinogen, which is cleaved to Ang I via renin secreted by juxtaglomerular glomeruli.^57^, ^58^ ACE is expressed by vascular endothelial cells in organs associated with the circulatory system and converts Ang I to Ang II.^59^, ^60^ Ang II is one of the major effector molecules in the RAS pathway and exerts its function by activating the following G protein‐coupled receptors: AT1R and AT2R.

AT1R and AT2R are two distinct subtypes of receptors that bind to Ang II, but they exhibit different affinities for Ang II.^61^, ^62^, ^63^, ^64^ AT1R is widely distributed in various tissues and organs, including the heart, blood vessels, kidneys, and brain.^65^ When Ang II binds to AT1R, it results in vasoconstriction, inflammation promotion, and increased blood pressure, among other effects. Therefore, AT1R is regarded as a proinflammatory and prohypertensive receptor. In contrast, AT2R is primarily expressed in fetal tissues but also in some adult tissues such as the brain, blood vessels, and heart.^65^ When activated, AT2R results in vasodilation, anti‐inflammatory effects, and reduces blood pressure. Hence, AT2R is considered an anti‐inflammatory and antihypertensive receptor. In summary, AT1R is linked to harmful effects on the cardiovascular system, while AT2R is associated with protective effects.

Ang II/AT1R is the most central regulatory signaling of the RAS, and its slight changes cause vasoconstriction and vasodilation, which regulate blood pressure. In 1999, Daugherty and Cassis found that Ang II promotes the development of atherosclerosis in low‐density lipoprotein receptor (LDL) knockout mice.^30^ The following year, they also found that Ang II promotes atherosclerosis development and AAA formation in apolipoprotein E (ApoE) knockout (−/−) mice.^30^ These two studies established an important foundation for subsequent studies on the role of Ang II in aortic lesions. Subsequent studies have identified the mechanisms by which Ang II causes the formation of AAA. Initially, it was believed that Ang II caused mechanical strain on the aortic wall through hypertension, leading to the formation of AAA. However, Ang II also stimulates the production of reactive oxygen species (ROS) and proinflammatory cytokines that contribute to the development of AAA. ROS can cause oxidative stress, leading to damage to the vascular endothelium and smooth muscle cells, resulting in the degradation of the extracellular matrix (ECM) and weakening of the aortic wall. Proinflammatory cytokines can activate MMPs, which degrade the ECM proteins, such as elastin and collagen, leading to the loss of arterial wall integrity and the formation of AAA. Moreover, Ang II can promote the migration and proliferation of inflammatory cells, including macrophages and T cells, into the aortic wall. These cells release additional cytokines and MMPs, resulting in further damage to the arterial wall and contributing to the development of AAA. Overall, Ang II plays a significant role in the pathogenesis of AAA by promoting vasoconstriction, oxidative stress, inflammation, and degradation of the ECM. It also indicates that targeting the Ang II pathway, particularly the AT1R receptor, may represent a potential therapeutic strategy for the prevention and treatment of AAA.

Recent years, many other molecular mechanisms have been identified and, as a result, many targets for therapeutic intervention have been derived. For example, Bian et al.^66^ found that HMGB1/TLR4‐mediated necrosis enhanced AAA development in an Ang II‐induced AAA mouse model. Whereas the necrosis inhibitor Nec‐1 and TLR4 blocker TAK‐242 attenuated Ang II‐induced AAA development while downregulating HMGB1/TLR4 expression. Similarly, Dai et al.^67^ found that Dexmedetomidine ameliorated Ang II‐induced endothelial cell dysfunction and vascular smooth muscle cell (VSMC) phenotypic transition by inhibiting the HMGB1/TLR4/NF‐κB signaling pathway through in vivo and in vitro experiments. Zhao et al.^68^ found in clinical studies and mouse studies that the endothelial *Ccni* gene plays a protective role in Ang II‐induced AAA by activating the Rb/E2f1/Dhfr signaling pathway that mediates coupling of eNOS and downregulation of ROS products. In an in vitro study of Ang II‐treated VSMCs, Chen et al.^69^ found that NF‐κB kinase subunit ε deficiency alleviated apoptosis and mitochondrial damage, while reducing autophagy. Whereas treatment with bafilomycin A1 reversed these results. In an Ang II‐induced AAA mouse model, Zhou et al.^70^ found that inhibition of protein phosphatase 2A (PP2A) accelerated AAA development by regulating ERK1/2 and NF‐κB signaling pathways, whereas activation of PP2A reduced AAA formation. In an ApoE(−/−) mouse study, Wang et al.^71^ found that Ang II upregulated the level of IL12p35. In turn, IL12p35 silencing exacerbated Ang II‐induced AAA formation by upregulating the levels of inflammatory factors IL‐1β, IL‐6, and TNF‐α, inducing apoptosis in VSMCs, as well as elevating STAT4 phosphorylation levels. STAT4 knockdown eliminated IL12p35‐mediated proinflammatory responses and apoptosis. Xie et al.^72^ found in a mouse model that Ang II exacerbated AAA progression by upregulating the CCL7‐mediated CCR1/JAK2/STAT1 signaling pathway to promote macrophage polarization toward M1. By contrast, CCR1 blockers, JAK2/STAT1 pathway inhibitors, and CCL7‐neutralizing antibodies all attenuated Ang II‐induced vascular remodeling. Lu et al.^73^ found that Ang II accelerated AAA formation by upregulating FoxO3a to promote VSMC phenotypic switching. Similarly, genes/proteins that have been shown to play a critical role in Ang II‐induced AAA formation include, but are not limited to, thrombospondin‐2, BAF60a, ventricular zone expressed PH domain containing 1, and STAT3.^74^, ^75^, ^76^, ^77^

RNAs are important cellular regulators, and recent studies have successively demonstrated their role in Ang II‐induced AAA formation. For example, Le et al.^78^ found that long‐stranded noncoding RNAs (lncRNAs) growth‐arrest‐specific transcript 5 induced VSMCs apoptosis and exacerbated AAA pathogenesis through activation of the zeste homolog 2‐mediated RIG‐I signaling pathway in an Ang II‐induced AAA mouse model. Hu et al.^79^ found that downregulation of microRNA (miRNA)‐218‐5P promoted the growth and migration of VSMCs and inhibited apoptosis, whereas upregulation of miRNA‐218‐5p suppressed the expression of IL‐8, IL‐1β, MMP‐9 and netrin‐1in THP‐1 cells. Yang et al. analyzed AAA‐related miRNAs and their target genes in humans and mice by microarray, and they found that downregulated miR‐145 may target RAC2 and downregulated miRNA‐30c‐2 may target PIK3CD, IL1B, and RAC2 to potentially affect AAA formation.^80^ Shi et al. demonstrated that Ang II may promote oxidized low‐density fatty acids (ox‐LDL)‐induced macrophage polarization toward M1 and release of proinflammatory evidence factors through downregulation of miRNA‐144‐5p‐mediated TLR2.^81^ In turn, miRNA‐144‐5p agonists reversed these results. These studies suggest that targeted therapy against multiple types of RNA may also be a potential approach to prevent AAA formation.

A disintegrin and metalloprotease with thrombospondin motifs (ADAMTS) is a family of extracellular proteases that are also involved in Ang II‐induced AAA formation.^82^, ^83^ A clinical study found that compared with normal arteries, AAA generally exhibited lower mRNA expression of *Adamts* such as *Adamts1, 4, 5, 8, 9, 10, 17*, and *Adamtsl1*.^84^ Subsequent investigations were carried out on ADAMTS1 in this study; however, the results were unexpected. In contrast to normal tissue, the expression levels of ADAMTS1 protein were higher in AAA, and it was mainly present in VSMCs and macrophages. However, ADAMTS1 overexpression in mice did not result any distinct arterial characteristics in an Ang II‐induced AAA mouse model. Hu et al.^79^ found that upregulation of miRNA‐218‐5p could mitigate the effect on apoptosis of VSMCs by targeting ADAMTS5. Li et al.^79^ found that miRNA‐126a‐5p may reduce ECM degradation by decreasing ADAMTS4 expression, thereby limiting Ang II‐induced AAA formation.^85^ These studies suggest that ADAMTS is involved in Ang II‐induced AAA formation, but the role of different subtypes of ADAMTS may be inconsistent. Therefore, targeted inhibition of the MMPs and ADAMTS family is considered to be the key to the treatment of AAA,^86^ but the mechanistic aspects still need further exploration.

### 4.3 The alternative RAS pathway ACE2/Ang‐(1–7)/MasR

In addition, the RAS has another axis the so called alternative pathway ACE2/Ang‐(1–7)/MasR. The discovery of ACE2 tool place in the early 2000s and was identified as a homologue of ACE.^87^, ^88^ Its primary function is to convert Ang I to Ang‐(1–9) or Ang II into Ang‐(1–7).^88^ This conversion helps to counterbalance the effects of the classical RAS pathway; in other words, it decreases the level of Ang II while increasing the level of Ang‐(1–7).^89^, ^90^, ^91^ The discovery of ACE2 and its role in regulating the RAS has led to significant advancements in our understanding of cardiovascular physiology, as well as its implications for various diseases, including hypertension, heart failure, and, more recently, its role as the entry receptor for the SARS‐CoV‐2 virus, the causative agent of COVID‐19.^92^ This discovery has also opened up new avenues for potential therapeutic interventions targeting the RAS and ACE2 for the treatment of cardiovascular and other related disorders.

Several recent studies have demonstrated that ACE2 can reduce the formation and severity of Ang II‐induced AAA.^93^, ^94^, ^95^, ^96^ Furthermore, diminazene aceturate, an ACE2 activator, has a similar function in significantly increasing ACE2 expression and activity, leading to elevated plasma Ang‐(1–7) levels.^96^ Animal studies have also indicated that DIZE can mitigate Ang II‐induced AAA formation and progression.^96^ Despite numerous investigations on DIZE activating ACE2/Ang‐(1–7)/MasR, its specific role in AAA remains limited. There are also studies demonstrating that overexpression of ACE2 or infusion of recombinant ACE2 can improve vascular dysfunction and atherosclerosis, but there are no studies on AAA.^97^, ^98^, ^99^, ^100^, ^101^, ^102^ This section highlights key studies on the correlation of ACE2/Ang‐(1–7)/MasR with AAA (Table 1). The discussion on the involvement of Ang‐(1–7)/MasR in AAA formation will be continued in the following paragraphs.

**TABLE 1:** Major studies of correlations between alternative RAS axis and AAA.

No.	Main research contents	Level of evidence	References
1	ACE 2 and AAA formation	Clinical observations and transgenic mice studies	^93^, ^94^, ^95^, ^96^
2	MasR and AAA formation	Transgenic mice studies	^22^, ^24^, ^103^
3	Ang‐(1–7) and AAA formation	Transgenic mice studies	^104^

The heptapeptide Ang‐(1–7) can be converted from ACE2/ACE/neprilysin cleavage of Ang II or endopeptidase cleavage of Ang I. Next, Ang‐(1–7) can bind to the MasR and counteract most of the harmful effects of the ACE/Ang II/AT1R, especially in some specific disease processes.^105^, ^106^ Previous studies showed that Ang‐(1–7) barely improved impaired vascular physiology and atherosclerosis progression after deletion of MasRs, suggesting that MasRs are the primary receptors for Ang‐(1–7).^23^, ^107^

**FIGURE 4:**
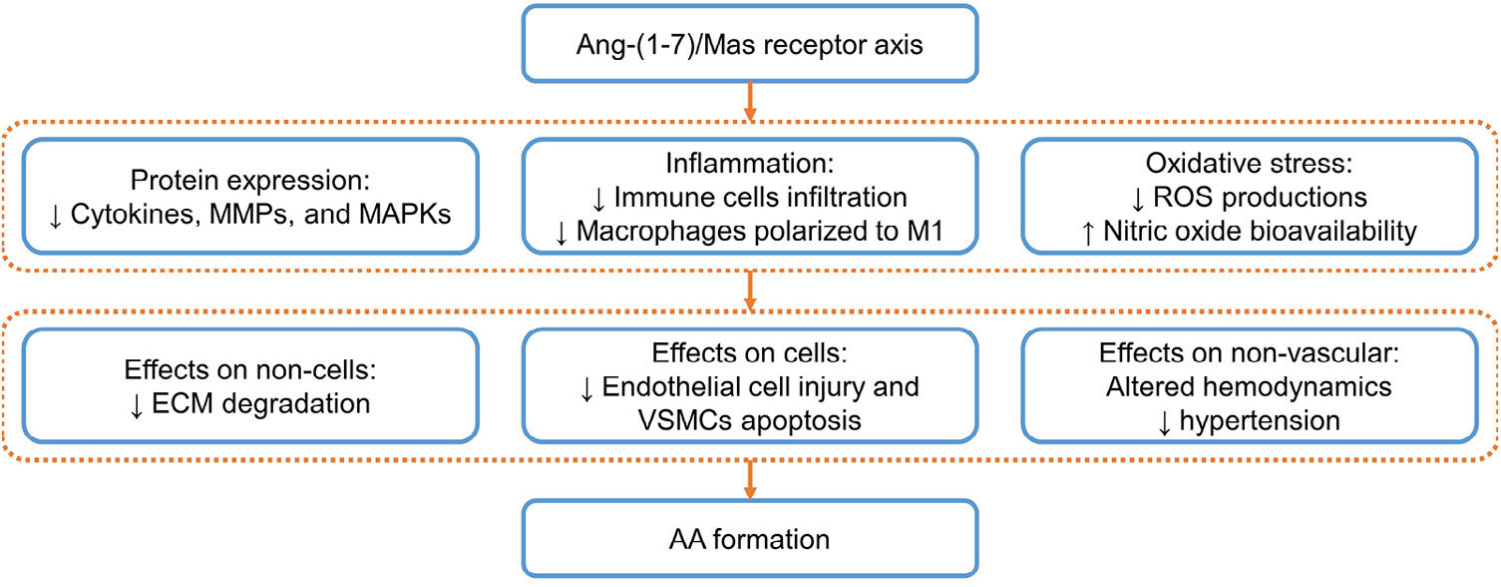
Activation of the Ang‐(1–7)/MasR axis attenuates vascular injuries and related complications by decreasing inflammation, oxidative stress, and the expression of inflammatory cytokines, MMPs, and MAPKs, leading to a reduction in the formation of AA. MAPK, mitogen‐activated protein kinase.

The activation of the Ang‐(1–7)/MasR axis has proven to be effective in reducing vascular‐related diseases, such as vascular dysfunction, atherosclerosis, and aneurysms (Figure 4). This is achieved by reducing oxidative stress, increasing nitric oxide bioavailability, and reducing inflammation.^20^, ^21^, ^22^, ^23^, ^24^ Conversely, when MasRs are knocked out, the protective effects of Ang‐(1–7) are abolished, and Ang II‐induced vascular injuries may even worsen.^22^ Furthermore, MasR deficiency promotes macrophage migration and infiltration and facilitates their polarization toward the proinflammatory phenotype M1, which exacerbates inflammatory progression.^20^ These are the more general pathological mechanisms, but as research continues, more molecular mechanisms are being discovered. Xue et al.^104^ discovered that Ang‐(1–7) has the ability to reduce the mRNA expression of *Il‐6*, *Tnf‐α*, and *Ccl2* in macrophages, inhibit VSMCs apoptosis and *Mmp‐2* expression, and stimulate MasR and AT2R to suppress the ERK1/2 signaling pathway. Their in vivo and in vitro experiments involving Ang II‐induced models showed that these actions of Ang‐(1–7) ultimately lead to a decrease in Ang II‐induced AAA and cell injuries. Ma et al. discovered that the activation of MasRs by AVE0991 and Ang‐(1–7) resulted in increased smooth muscle cells, along with decreased accumulation of macrophages, expression levels of *Mcp‐1* and *Tnf‐α*, and activity and expression of *Mmp‐2* and *‐9* in both Ang II‐treated models mouse AAA and human VSMCs.^103^ They further suggest that these effects may be linked to MasR activation‐induced reduction in oxidative stress and the downregulation of P38 and ERK1/2 signaling pathways.

## 5 THE ROLE OF RAS IN RISK FACTORS INDUCING AAA FORMATION

The role of RAS in regulating AAA formation is well known, but there are multiple risk factors that can initiate or influence the development of AAA. These risk factors can be modulated by RAS or in conjunction with RAS. The Ang II/AT1R axis is of particular interest, as it directly regulates vascular physiological functions such as immune response, blood pressure, and vasoconstriction. The relationship between these risk factors and vascular injuries related to RAS is not yet extensively studied. This section will explore the association between high‐risk factors for AAA and RAS, providing a broader understanding of RAS's role in AAA formation.

### 5.1 Age

There are many risk factors for the development and incidence of AAA, including but not limited to smoking, cardiovascular disease, genetics, and specific etiologies. However, it is accepted that age is an important factor in AAA formation, with the incidence of AAA in younger populations being relatively rare.^3^, ^17^ A study based on a Pakistani population showed that the diameter of the infrarenal aorta was positively correlated with age and the diameter of the infrarenal aorta was smaller in women than in men.^108^ Similarly, in the populations with Marfan syndrome and Loeys‐Dietz syndrome, the diameters of the aortic arch, isthmus, and abdominal aorta are positively associated with age.^109^

Enlargement of the abdominal aorta can be caused by degenerative organ diseases, underlying conditions, and hypertension. These factors are closely linked to elevated oxidative stress, chronic low‐grade inflammation, and immune dysregulation.^110^, ^111^, ^112^, ^113^ Aging is associated with increased oxidative stress in the vascular system due to factors such as the accumulation of ROS, decreased antioxidant defenses, and changes in blood vessel structure and function. ROS can damage endothelial cells, leading to inflammation, impaired vascular function, and an increased risk of cardiovascular disease. Aging is also associated with a decline in nitric oxide production, which helps regulate blood vessel tone and protect against oxidative stress.^114^, ^115^ Additionally, aging can contribute to vascular inflammation, which can lead to the development of various cardiovascular diseases, through mechanisms such as oxidative stress, telomere shortening, and the accumulation of senescent cells.^116^, ^117^, ^118^ Vascular inflammation is a complex process that involves the activation of immune cells, such as macrophages and T cells, and the release of proinflammatory molecules, such as cytokines and chemokines. These molecules can damage the lining of blood vessels, making them more susceptible to plaque buildup and artery narrowing. As a result, vessels can accumulate lesions, leading to endothelial injury, VSMCs apoptosis, elastic fiber rupture, collagen degradation, and eventually expansion under the impact of blood pressure until they rupture. Overall, reducing oxidative stress and inflammation in the vascular system may have important implications for promoting healthy aging and preventing vascular disease.

Recent studies suggest that age‐related changes in RAS expression levels may be associated with aortic lesions.^119^ The Ang II/AT1R axis and Ang‐(1–7)/MasR axis are discussed separately. With regards to Ang II/AT1R, aging typically leads to a decrease in renin and Ang II levels, and an increase in AT1R expression levels.^120^, ^121^, ^122^, ^123^ Although age does not affect AT1R sensitivity,^124^ the downregulation of Ang II levels may be a compensatory mechanism to counteract the effects of AT1R overexpression. There is also another possibility, the upregulation of AT1R may be a compensatory mechanism for the body to cope with Ang II downregulation, which facilitates the maintenance of a more stable blood pressure. Without considering changes in Ang II levels, age‐induced AT1R overexpression may be a significant contributor to aortic lesions.

There is limited research on the effects of aging on Ang‐(1–7)/MasR, but available studies indicate similar trends in the expression levels of Ang‐(1–7)/MasR and Ang II/AT1R. With increasing age, the expression levels of Ang‐(1–7) decrease in the brain while the expression levels of MasRs increase in the retina.^125^, ^126^ However, there is a lack of data on older age groups. A recent study investigated the role of MasRs in aging‐related vascular repair dysfunction and found that partial or total inhibition of MasR expression may lead to accelerated vascular aging through oxidative stress and low NO bioavailability.^127^ In contrast, in nontransgenic mice, increasing age did not cause vascular repair dysfunction. This suggests that MasRs play a protective role in preventing vascular senescence and injury, and that the age‐induced increase in MasR expression may represent a self‐protective mechanism of the vasculature. Conversely, decreased Ang‐(1–7) expression may serve as a balancing mechanism for increased MasR expression and vice versa.

We propose that gene expression balance is crucial in maintaining homeostasis in the body, particularly in relation to the impact of aging on the RAS. Aging often leads to downregulation of Ang II and upregulation of AT1R. However, if Ang II is not appropriately downregulated, AT1R can become overactivated, requiring treatment with AT1R inhibitors. Additionally, the balance between AT1R and MasR is essential, as overexpression of MasRs can antagonize AT1R, feedback Ang‐(1–7) reduction, and promote vascular protection. When the AT1R/MasR ratio is elevated, it can cause vascular injury. As a result, several studies have used the ratio to assess RAS expression levels.

Ageing causes alterations in peptides and receptors of the RAS, such as expression levels, activity, and affinity. Existing preclinical studies are mostly based on young and middle‐aged animals and are insufficient to mimic the physiology of people over 65 years of age. Whether the cause of AAA incidence in the elderly population is related to Ang‐(1–7)/MasR or therapeutic modalities targeting Ang‐(1–7)/MasR apply to the elderly population remain worthy of further exploration.

### 5.2 Smoking

It is well recognized that smoking is an important exogenous risk factor for accelerated AAA formation, expansion, and rupture. Moreover, smoking is a high‐risk factor for AAA in both men and women,^128^, ^129^ and women's vascular may be more susceptible to tobacco than men's.^130^, ^131^, ^132^ A Swedish study showed that 87% of patients had experience AAA due to smoking.^133^ Even with the people that had quit smoking, the odds of AAA were higher than in the nonsmoking group.^134^, ^135^ In addition, multiple studies have shown that the incidence of AAA is positively related to the dose and duration of smoking exposure.^134^, ^136^

There are many studies on the mechanisms of smoking‐induced AAA formation, and it has been characterized that ingestion of substances from tobacco leads to the upregulation of inflammatory factors, disruption of T‐cell responses, epigenetic modifications of genes, and overexpression of MMPs and elastases.^135^ The direct consequences are endothelial dysfunction, impairment of VSMCs, loss and dysfunction of parenchymal cells, and structural remodeling of ECM.

In addition, recent studies have also found that tobacco influences the expression levels of RAS peptides. In other words, tobacco may cause vascular injury by activating the Ang II/AT1R axis, which ultimately leads to AAA formation. A clinical study showed that compared with healthy and coronary populations, smokers had significantly higher levels of serum ACE, oxidative markers malondialdehyde, ox‐LDL, total antioxidant capacity, high‐sensitivity C‐reactive protein, and MMP‐9.^137^ To put it in other words, smoking increases levels of inflammation and oxidative stress in the body, as well as upregulating ACE levels. We presume that elevated levels of ACEs may preferentially cause an increase and accumulation of Ang II levels and lead to a further increase in blood pressure and vascular injury. The question arises as to whether ARBs can be used to alleviate smoking‐induced vascular injury, and a previous study provides a definitive answer. In this study, patients with essential hypertension and high plasma renin activity were treated with irbesartan, an ARB, and the results showed that smokers had a significant improvement in vascular function (carotid‐radial pulse wave velocity) compared with nonsmokers.^138^ This suggests that ARBs treatment may benefit hypertensive chronic smokers with elevated plasma renin levels by reducing arterial stiffness and wave reflection. Another animal study yielded comparable findings, indicating that prolonged exposure to tobacco led to elevated levels of Ang II and ACE expression, while reducing ACE2 expression in the lungs.^139^ However, the administration of losartan, an ARB, was able to reverse these effects. Hence, it is plausible that smoking may result in an excessive build‐up of Ang II due to the upregulation of ACE/Ang II and the downregulation of ACE2. This, in turn, could trigger vascular inflammation, oxidative stress, and increased proteases by activating AT1R, ultimately leading to vascular injury.

Furthermore, the cascade effect of Ang II/AT1R may be exacerbated by the age‐related elevation of AT1R levels and the accumulation of Ang II due to various other factors. Previous preclinical studies have demonstrated that cigarette smoking exacerbates cardiovascular diseases and AAA formation induced by Ang II,^140^, ^141^ providing evidence that the effects of smoking and Ang II can be compounded. Stolle et al. discovered that while smoke exposure or Ang II treatment alone had minimal effects on *Mmps* expression levels in ApoE(−/−) mice, the combined exposure significantly upregulated the gene expression of *Mmp‐2*, *‐3*, *‐8*, *‐9*, and *‐12* in abdominal aorta, leading to accelerated AAA formation and severity.^140^ Considering that the main source of MMPs is macrophages, we speculate that smoking causes a rise in MMPs by promoting inflammation. Additionally, according to a study conducted on mice by Fried et al.,^141^ chronic nicotine inhalation was found to prevent Ang II‐induced left ventricular posterior wall thickening and induce a shift in cardiac remodeling from concentric to eccentric hypertrophy. Unfortunately, this study did not explore the mechanism in depth. Overall, we suggests that smoking accelerates the formation and progression of AAA by promoting ACE/Ang II expression and inhibiting ACE2/Ang‐(1–7) expression.

### 5.3 Genes

Many genes have been shown to influence the formation of AAA in human. For example, mutations in TGF‐β family (TGFBR1, TGFBR2, TGFB1, and TGFB3) lead to AAA formation.^142^, ^143^, ^144^, ^145^, ^146^ However, further research has been conducted on this gene in mice, and interesting results have been obtained.^147^ It was found that the role of TGF‐β signaling in the formation of AAs depends on the tissue location. Systemic TGF‐β blockade has increased the severity of AAA, adventitial thickening, and macrophage infiltration. In contrast, deficiency of smooth muscle‐specific TGF‐β signaling only reduced the thickness of the middle layer of the abdominal aorta. Additionally, in the same mouse, the deficiency of smooth muscle‐specific *Tgfbr2* accelerated thoracic aortic lesions. This study suggests that external signals of smooth muscle protect the abdominal aorta, while internal signals protect the thoracic aorta. This study further emphasizes the tissue specificity of genes in regulating aortic pathology and the importance of targeted therapy.

Similarly, mutations in the signal transduction protein SMAD3 result in the occurrence and development of various types of arterial aneurysm diseases through causing vascular remodeling, collagen fiber recombination, and inflammatory cells infiltration.^148^, ^149^, ^150^ Although it has been confirmed that SMAD3 mutations combined with RAS disorders can accelerate AAA formation, there has been a controversy about the correlation between the two factors. One study suggests that tissue fibrosis caused by TGF‐β/SMAD2/3 may be independent of RAS.^151^ In another cell study, Ang‐(1–7) was found to downregulate TGF‐β1/SMAD2/3 and inhibit the transformation of human lung fibroblasts into myofibroblasts by reducing the expression of Collagen I.^152^ Unfortunately, these studies were mainly based on other tissues and cells, and not on AAA or blood vessels. Therefore, the TGF‐β/Smad3 pathway and RAS may be parallel relationships, but they can also affect each other's expression.

MYH11 is a protein known as smooth muscle myosin heavy chain, which plays a vital role in the contraction and relaxation of smooth muscle cells. Clinical studies have demonstrated that mutations in the *MYH11* gene can significantly increase the likelihood of developing AAA, especially in those with a family history of the condition.^153^, ^154^ Such mutations can impair the function of smooth muscle cells in the wall of the aorta, leading to the formation of aneurysms. Recent studies have found that while Ang II/AT1R axis activation‐induced vascular remodeling is independent of the vascular disease caused by the *MYH11* gene, the cooccurrence of these two factors can accelerate the process of vascular remodeling.^155^ Therefore, it is possible that when treating AAA, targeting both MYH11 and RAS may be necessary for optimal management and prevention of disease progression.

Although there have been some gene mutations linked to AAA, there has been relatively little research on this topic, and we did not provide a more detailed description here. For instance, MMPs are widely recognized as key proteases involved in AAA formation, but we could not find any clinical reports linking MMP mutation to AAA formation. Moreover, *JAK2*V617F is the most common driving mutation in myeloproliferative neoplasms, which can accelerate Ang II‐induced AAA formation by promoting macrophage infiltration and the secretion of *Mmp‐2*, *‐9*, and *‐13*.^156^ Similar genes also include, but are not limited to, *MYLK*, *ACTA2*, *MAT2A*, and *LOX‐1*.^157^, ^158^, ^159^, ^160^, ^161^ According to these studies, it is believed that these genes may not necessarily drive RAS, but they may have a synergistic effect with RAS in promoting AAA formation. As a result, future treatments for this type of AAA may require a combination of gene‐targeted therapy drugs and RAS regulatory drugs. In other words, Ang‐(1–7)/MasR is likely to be a target for synergistic therapy.

### 5.4 Marfan and Loeys‐Dietz syndromes

Marfan and Loeys‐Dietz syndromes are hereditary diseases that can accelerate the formation of AAA.^109^ The underlying pathological mechanisms of both syndromes are not fully understood, but they are known to be connective tissue disorders caused by autosomal dominant disorder‐mediated TGF‐β signaling.^162^, ^163^ Basic studies for both genetic diseases have shown that inactivation of TGF‐β signaling downregulates Smad signaling and enhances MAPK signaling (e.g., P38 and ERK1/2), thereby promoting the release of MMP‐2, ‐9, and ‐12 and the increase of inflammation in the aorta.^164^, ^165^, ^166^, ^167^ Wang et al.^168^ found that neutralizing TGF‐β enhanced monocyte accumulation and MMP‐12 activity, whereas monocyte depletion or MMP‐12 deficiency inhibited AAA progression. These results suggest a critical role for TGF‐β in AAA formation and inflammatory cells regulation in Marfan syndrome mice model. In addition, these hereditary diseases are associated with enhanced Ang II and its associated signaling pathways, which can lead to ECM remodeling, inflammatory cytokine and chemokine expression, aortic wall inflammatory cell infiltration, VSMCs apoptosis, and endothelial injuries.^169^ A study of these patients' plasma showed that they all had significantly increased levels of the ECM remodeling biomarker MMP‐9, and that Loeys‐Dietz syndrome patients also had significantly increased levels of the inflammatory markers pentraxin 3 and CD25+ T cells.^170^ These studies further indicate the close association of TGF‐β, Ang II/AT1R and inflammation in both syndromes.

Interestingly, inflammation and vascular wall remodeling are also typical symptoms of Ang II/AT1R overactivation. Several therapeutic studies targeting anti‐inflammatory and antioxidative stress have therefore been developed.^169^ Anti‐inflammatory drugs and antioxidants attenuate exacerbation of aortic lesions in mice with Marfan syndrome. For example, Guido et al.^171^ discovered that administering methotrexate decreased the levels of CD68 macrophages, CD3 T lymphocytes, TNF‐α, caspase‐3, type 1 collagen, TGF‐β, ERK1/2, and SMAD3 protein expression in a mouse model of Marfan syndrome. This treatment effectively slowed down the progression of aortic dilation. However, some studies hold a different view that suppressing AAA development by inhibiting inflammatory signaling had little effect. A clinical study lasting 2 years showed that doxycycline did not significantly reduce the development of small infrarenal AAAs.^172^ Similarly, Franken et al.^173^ discovered that the anti‐inflammatory drugs methylprednisolone (corticosteroid) and abatacept (T‐cell‐specific inhibitor) do not reduce the rate of aortic dilation in Marfan mice, but may instead increase aortic injury. Meanwhile, they suggested that the most promising drug is losartan (an AT1R antagonist) thus inhibiting phosphorylated Smad2 signaling.

Theoretically, targeting downstream signaling pathways of TGF‐β and Ang II, such as ERK1/2, mTOR, PI3/Akt, P38/MAPK, and Rho kinase signaling pathways, could attenuate the pathogenesis of AAA. However, clinical drugs targeting TGF‐β have all failed, possibly due to the cell‐specific regulation of aortic lesions by TGF‐β. For example, external signals from smooth muscle protect the abdominal aorta, while internal signals protect the thoracic aorta.^147^ Similarly, studies using MAPK inhibitors to inhibit AAA have not met expectations, highlighting the need for targeted drug delivery. Although challenging, targeted drug delivery may hold the key to treating AAA, with drugs targeting the RAS receiving increasing attention.

The above discussion suggests that it seems difficult to achieve inhibition of AAA development with TGF‐β or anti‐inflammatory related drugs and that drugs targeting RAS may be a better option. It is now thought that the Ang II/AT1R axis may play an important role in the development of aneurysms caused by both syndromes as well. For example, AT1R antagonist/blockers may delay the progression of AAs in Marfan Syndrome.^174^, ^175^ A mice study showed that Losartan reduced heart rate, blood pressure, elastic fiber dissection, TGF‐β signaling, and phosphorylated Smad2 accumulation, thereby reducing AA formation.^174^ In the same experiment, propranolol (a beta‐adrenergic blocker) reduced the rate of aortic growth but did not prevent aortic remodeling and sustained dilation. A clinical study showed that both Losartan and Irbesartan significantly reduced the rate of progressive aortic root dilatation in Marfan syndrome patients.^175^ Similarly, either AT1R inactivation or Losartan administration alleviated aortic diameter expansion in Loeys‐Dietz syndromes.^176^, ^177^ A mouse study conducted on Loeys‐Dietz syndrome demonstrated that the dilation of the aortic root diameter could be reduced by attenuating both systemic and local AT1R signaling.^176^ This was achieved by decreasing the phosphorylation levels of Smad2/3 and ERK. Also, this study suggests that inhibiting local AT1R signaling is crucial for enhancing the architecture of aortic tissue. However, the best therapeutic outcomes can be achieved by simultaneously inhibiting both systemic and local AT1R signaling. These studies seem to suggest that inhibition of Ang II/AT1R activity can contribute to the inhibition of aortic diameter expansion.

Despite showing promising results in animal experiments, the drugs under investigation have not yielded positive outcomes in previous randomized controlled trials during clinical studies.^178^, ^179^, ^180^, ^181^ However, a recent clinical study conducted on patients with Marfan syndrome has provided new insights.^182^, ^183^ The researchers performed a comprehensive meta‐analysis incorporating a substantial amount of historical data, with a particular focus on numerous subgroups. Notably, they utilized body surface area as the primary endpoint. The study concluded that the utilization of ARBs alone, β‐blockers alone, or a combination of both significantly reduced the rate of increase in *Z*‐scores in the aortic root by approximately half. This finding offers a glimmer of hope for the potential efficacy of these drugs in treating AAAs. However, it is important to note that future investigations should consider conducting more detailed subgroup studies to thoroughly assess the effectiveness of these treatments.

Collectively, both syndromes demonstrate a close association with the activation of the Ang II/AT1R axis. Notably, inhibiting this axis has shown significant promise in mitigating disease progression. However, it is important to highlight that inhibiting downstream signaling pathways alone does not seem to effectively achieve the desired therapeutic outcome. Another potential avenue for therapeutic intervention lies in the Ang‐(1–7)/MasR axis, which antagonizes the efficacy of the Ang II/AT1R axis, making it a promising target for intervention. The efficacy of the Ang‐(1–7)/MasR axis is demonstrated by a study employing a muscle regeneration model in mice with Marfan syndrome. It revealed that Ang‐(1–7) administration effectively alleviated muscle fibrosis and improved both satellite cell dysfunction and muscle contractile function.^184^ This showcases the potential of the Ang‐(1–7)/MasR axis in ameliorating the symptoms of both syndromes. However, it is worth noting that there is currently a paucity of research investigating the role of the Ang‐(1–7)/MasR axis inAAA associated with these syndromes. Consequently, further exploration in this direction holds great promise and should be pursued diligently.

### 5.5 Infection

Infection‐induced AAA, also known as infected native AA (INAA), is a much rarer type of aneurysm. A Swedish statistic shows that INAA occurs in less than 1% of AAA, 60% in the suprarenal and 40% in the infrarenal.^185^, ^186^, ^187^, ^188^ The most common sources of infection in INAA are *Salmonella* (33.4%), *Staphylococcus* (15.6%), and *Streptococcus* (10.4%), while *Escherichia coli* (3.1%), fungi and viruses are less common.^9^ Besides conventional surgical treatment, studies suggested that additional antibiotic therapy is required. The rarity of INAA has led to a lack of early sampling and diagnostic methods, and in many cases, conventional AAA therapy is still used. Even with culture testing, 25−33% of results may be negative, leading to a misdiagnosis.^187^ There is also a lack of in‐depth studies on the timing and duration of antimicrobial therapy. In addition, patients with atherosclerosis or aneurysms are more likely to be infected.^187^ This also suggests that such patients need other coupling treatments in addition to the conventional surgical and antimicrobial treatments.

Recently, a case of COVID‐19‐induced multiple cerebral aneurysms in a 55‐year‐old patient without any detectable aneurysms prior to infection has been reported.^189^ The researchers suggest that overactivated inflammation and RAS may be the pathophysiological mechanism of these multiple cerebral aneurysms. Inflammation is known to be a normal immune response to infection, where monocytes are recruited to the injury site and differentiate into a proinflammatory phenotype of macrophages. Activated macrophages secrete MMPs that will degrade the ECM, which in turn leads to vascular remodeling. Therefore, we hypothesize that INAA formation may be associated with inflammation and RAS; however, critical evidence is still lacking.

### 5.6 Gut microbiome

The gut microbiome is also thought to be a key factor in inducing AAA. Differences in gut microbiota were found in both AAA mouse models and AAA patients compared with normal populations.^190^, ^191^ Moreover, a variety of gut‐related flora, such as *Streptococcus*, *Campylobacter*, and *Campylobacter urealyticus* are found in aneurysm walls and intravascular plaques, which may be caused by intestinal bacterial translocation, infection, and blood‐borne transmission.^192^, ^193^, ^194^ It is believed that dysregulation of the gut microbiome promotes the formation and development of AAA by upregulating oxidative stress, activating inflammation, and releasing small molecule metabolites to exacerbate arterial remodeling.^195^, ^196^ Also, anti‐inflammatory factor intervention can prevent AAA rupture to some extent.^196^, ^197^

The modulation of the gut microbiome, which affects the local RAS, has recently garnered considerable attention within the realm of research. First, gut microbes have the capability to influence the local RAS through the production of certain metabolites, such as short‐chain fatty acids (SCFA) like propionic acid, butyric acid, and acetic acid.^198^, ^199^ These SCFA compounds can activate G protein‐coupled receptors, namely GPR41 and GPR43, thereby inhibiting the activation of Ang II/AT1R axis.^200^, ^201^ Consequently, this process leads to a decrease in blood pressure and a mitigating effect on the inflammatory response within the kidney. Second, gut microbes exert their influence on the local RAS by modulating the functionality of the gut barrier.^202^ The gut barrier acts as a safeguarding barrier, preventing the entry of detrimental substances and bacteria into the bloodstream. By preserving the integrity of the gut barrier, gut microbes minimize the permeation of endotoxins, which subsequently diminishes the activity of the local RAS. Furthermore, gut microbes possess the ability to modulate the local RAS through their influence on the immune system.^203^ Interactions between gut microbes and the host immune system have been observed, and several studies have demonstrated that gut microbes can impact the local RAS by regulating the differentiation and function of T cells. Specifically, gut microbes can influence the activity of the local RAS by maintaining the balance between regulatory T cells (Treg) and inflammatory T cells (Th17).^204^, ^205^, ^206^, ^207^ Treg cells exert inhibitory effects on the local RAS, while Th17 cells enhance its activity. In summary, gut microbes exert their influence on the regulation of the local RAS through the production of metabolites, modulation of gut barrier function, and modulation of the immune system. Importantly, dysregulation of the RAS may contribute to the development of AAA. These mechanisms of action not only enhance our understanding of the intricate relationship between gut microbes and localized AAA but also provide novel insights for future research and the development of therapeutic strategies.

In addition, some studies have shown that drug therapy is associated with the regulation of gut microbiome and RAS when improving aortic disease. For example, *Flavonifractorplautii* can prevents Ang II‐induced arterial stiffness in the mouse model. *Flavonifractorplautii* and its key effector molecule aconitic acid can prevent Ang II‐induced arterial stiffness in mouse model by inhibiting MMP‐2 activity, inhibiting local vascular inflammation, and reducing elastic fiber breakage.^208^ Moreover, Chai‐Gui decoction significantly reduced the blood pressure and arterial wall thickness in SHR rats, which may be related to the regulation of Lysophosphatidylcholine metabolism, RAS and the bacterial abundance of S24‐7 family in the intestine.^209^ Furthermore, a recent study has elucidated the connection between the gut microbiome and Ang‐(1–7). Aging‐induced dysbiosis of the gut microbiome causes inflammation and disrupts the gut barrier integrity by disrupting the ACE2/ACE balance. Interestingly, this process can be reversed through the sustained administration of Ang‐(1–7).^210^ These findings provide evidence for an interrelation between AAA, RAS, and the gut microbiome. In future endeavors, therapeutic interventions targeting aortic lesions may involve the modulation of the gut microbiome, administration of Ang‐(1–7), or a combination of both approaches, potentially offering an effective therapeutic modality.

### 5.7 Hypertension

Hypertension is a well‐established risk factor for AAA and has been extensively studied. Here, we provided a brief overview of the association between hypertension and RAS. First, hypertension exerts increased pressure on the arterial wall, potentially resulting in structural damage and heightened vulnerability of the vessel wall.^211^ Second, persistent hypertension contributes to the development of atherosclerosis, characterized by thickening and reduced elasticity of the vessel wall.^197^, ^212^, ^213^, ^214^ These changes render blood vessels more prone to dilation and rupture, thereby elevating the risk of AAA. Third, hypertension triggers inflammation and disruption of the vessel wall,^215^, ^216^ compromising the structural stability of the abdominal aortic wall and further increasing the likelihood of AAA formation. Last, hypertension induces damage to the intima‐media of blood vessels,^217^, ^218^ which initiates an inflammatory response and promotes platelet aggregation, thereby augmenting the risk of AAA. Consequently, hypertension amplifies the risk of AAA formation by exerting pressure on the arterial wall, fostering atherosclerosis, provoking inflammation and destruction of the vessel wall, and inducing intimal damage. These findings underscore the importance of effectively managing and controlling hypertension in the prevention of AAA formation.

The RAS plays a crucial role in the regulation of hypertension, functioning as an important hormonal regulatory system. It regulates various physiological processes including vasoconstriction, fluid volume regulation, and electrolyte balance. Ang II plays a significant role in elevating blood pressure through mechanisms such as increased vasoconstriction, enhanced sodium and water retention, and stimulation of aldosterone secretion.^219^ Imbalance or hyperactivity of the RAS contributes to the development and progression of hypertension. Consequently, inhibiting the activity of the ACE/Ang II/AT1R axis, achieved through the use of ACE inhibitors or ARBs, effectively reduces blood pressure and controls the development of hypertension.^220^ These medications act by blocking the production or action of Ang II, resulting in decreased vasoconstriction, reduced sodium and water retention, and ultimately lowering blood pressure levels. Therefore, interventions targeting the RAS represent a key therapeutic approach for managing hypertension and are also considered effective in preventing the progression of AAA.

The ACE2/Ang‐(1–7)/MasR axis assumes a critical role in the management of hypertension. First, ACE2 exerts a blood pressure‐lowering effect by attenuating vasoconstriction and fluid retention through the downregulation of Ang II. Second, Ang‐(1–7), upon binding to MasRs, elicits various beneficial effects such as vasodilation, anti‐inflammatory properties, and antifibrotic effects, thereby contributing to the reduction of blood pressure and improvement of vascular function. Furthermore, ACE2 and Ang‐(1–7) can effectively lower blood pressure by inhibiting RAS activity, consequently reducing the production and action of Ang II. Consequently, the ACE2/Ang‐(1–7)/MasR axis plays a pivotal role in ameliorating hypertension, leading to blood pressure reduction and enhanced vascular function. This suggests that targeting the ACE2/Ang‐(1–7)/MasR axis may hold promise as a potential therapeutic approach for the treatment of AAA.

### 5.8 Summary

Clinical practice has shown that ARBs or ACE inhibitors yield positive outcomes in the treatment of cardiovascular diseases, although long‐term use may lead to a side effects. We think that the role of hormones in maintaining normal physiological functions is not solely dependent on their absolute concentration but also on the balance between interacting hormones. Several studies have investigated the RAS using relative expression levels. In other words, when addressing cardiovascular diseases, it is important to not only focus on inhibiting the ACE/Ang II/AT1R axis but also consider activating the ACE2/Ang‐(1–7)/MasR axis.

From the above discussion, it is revealed that these high‐risk factors for AAA are correlated with upregulation of ACE/Ang II/AT1R axis and downregulation of ACE2/Ang‐(1–7)/MasR axis, suggesting that both axes may be therapeutic AAA targets. Currently, there are many drugs targeting the ACE/Ang II/AT1R axis, but fewer therapeutic modalities targeting the ACE2/Ang‐(1–7)/MasR axis. Several antagonists and agonists targeting MasRs have been developed, and related studies and evaluations are still ongoing. Additionally, directly use Ang‐(1–7) as a drug is also an interesting research direction. As an easily degradable short peptide, choosing the appropriate drug delivery method is crucial. Moreover, the Ang‐(1–7)/MasR axis as a therapeutic target is not applicable to the treatment of all AAAs. The potential side effects of drugs are not negligible in drug testing, as it will be described in the following sections. Next, we will focus on the research advances and future directions of Ang‐(1–7)/MasR as a therapeutic target and discuss the potential risks.

## 6 AGONISTS OF MasR

In this section, we will present research advances in MasR agonists, especially their efficacy and mechanisms in the prevention of aortic lesions.

### 6.1 Ang‐(1–7)

The heptapeptide Ang‐(1–7) is the most classical ligand of MasR. Previous studies mostly focused on Ang‐(1–7) in improving vascular dysfunction and atherosclerosis.^20^, ^22^, ^23^ Recent studies also found that Ang‐(1–7) exhibited good inhibitory effects on a variety of aneurysms, such as intracranial aneurysms, aneurysmal subarachnoid hemorrhage, TAA, and AAA.^22^, ^104^, ^221^, ^222^, ^223^, ^224^ A systematic study was also carried out by Xue et al.^104^ The in vivo results showed that Ang‐(1–7) inhibited the incidence and progression of Ang II‐induced AAA by suppressing macrophage infiltration, inhibiting the expression of the inflammatory factors IL‐6, TNF‐α, CCL2 and the protease MMP‐2, and reducing the apoptosis of VSMCs. However, the beneficial results of Ang‐(1–7) were both reversed after the administration of MasR antagonist A779 or AT2R antagonist PD123319. In vitro experiments showed similar results that Ang‐(1–7) not only inhibited the expression of proinflammatory factors induced by Ang II in macrophages, but also suppressed apoptosis and MMP‐2 expression in Ang II‐treated VSMCs. This suggests that Ang‐(1–7) has a protective effect on both the immune and cardiovascular systems by reducing the inflammatory response and preventing cell death and tissue damage.

### 6.2 AVE0991

AVE0991 is a nonpeptide compound that binds to the aortic endothelial cell membrane and exerts effects similar to those of Ang‐(1–7). Studies have shown that both AVE0991 and Ang‐(1–7) promote the release of NO and O_2_ from endothelial cells, and that AVE 0991 induces approximately five times the amount of NO release than that induced by Ang‐(1–7).^225^ A subsequent mouse study showed that Mas deficiency reversed the antidiuretic effect of AVE0991 during water loading, suggesting that AVE0991 exerts its biological effects through the activation of MasRs.^226^ Subsequent studies have successively confirmed the effects and mechanisms of AVE0991 in vascular protection, such as avoiding ischemia‐induced heart failure, alleviating asthma‐induced pulmonary vascular injury, reducing chronic neuroinflammation, attenuating heatstroke‐induced visceral injury, promoting vasodilation, and antiatherosclerosis.^227^, ^228^, ^229^, ^230^, ^231^, ^232^, ^233^, ^234^, ^235^ The mechanisms involved can be attributed to antioxidant, anti‐inflammation, restoration of mitochondrial function, antiapoptosis, and antipyroptosis.

AVE0991 is less studied in aneurysms. In 2020, Ma et al.^103^ proved that AVE0991 reduced Ang II‐induced AAA formation in ApoE(−/−) mice by decreasing inflammation and oxidative stress, inhibiting MMP‐2 and ‐9 expression, downregulating P38 and ERK1/2 signaling pathways, and antiapoptosis of VSMCs. However, another study showed that coadministration of the ACE inhibitor captopril with AVE0991 did not reverse transverse aortic constriction‐induced aortic expansion and lead to abnormal aortic remodeling.^233^ This illustrates that AVE0991, while slowing arterial expansion may still have a limited therapeutic effect.

### 6.3 AR234960

AR234960 is a recently discovered MasR agonist. Two in vitro studies have shown interesting results, which may indicate a new function after MasR activation. One study showed that AR234960 can upregulate connective tissue growth factor expression and promote collagen synthesis by activating the ERK1/2 signaling pathway in human cardiac fibroblasts.^236^ Another in vitro study based on VSMCs showed that AR234960 significantly increased cytosolic calcification,^237^ which may not be good for cells. In addition, AR234960 slightly elevated alkaline phosphatase activity as well as the expression levels of OPG, OPN, BMP‐2, and Collagen II; however, no significant difference (*p* ≥ 0.05) was found. Cardiac fibroblasts are cells that play a crucial role in maintaining the structural integrity and function of the heart. These cells can secrete ECM proteins and cytokines that contribute to tissue remodeling and repair. VSMCs are cells that make up a significant portion of the arterial wall and play a key role in regulating blood vessel tone and structure. Collagen is the main structural protein of ECM and has an important function in maintaining tissue structural integrity and elasticity.^238^, ^239^, ^240^, ^241^ These studies all pointed out that MasR activation promotes collagen synthesis. It is known that inflammation promotes collagen and ECM degradation by upregulating the MMPs activity, thus reducing tissue firmness and elasticity. Additionally, aging also reduces the amount of collagen in tissues, which makes tissues lose elasticity.^242^ Therefore, activation of MasR‐induced collagen synthesis may be a compensatory mechanism and may reinforce tissue firmness to a certain extent.

Currently, much of the existing research on the role of Ang‐(1–7)/MasR in AAA formation has focused on the involvement of inflammatory cells, such as macrophages and T cells. However, the two articles highlight the potential importance of MasR activation in other cell types, such as cardiac fibroblasts and VSMCs. Overall, these studies suggest that MasR activation could have a broader impact beyond its effects on inflammatory cells in AAA formation. Further research is needed to fully understand the role of MasR in these different cell types and how it may influence the AAA development and progression. Nevertheless, existing studies are limited to cellular investigations only and have not yet involved in vivo experiments. In any case, it suggests that the use of AR234960 for the activation of MasRs or treatment of aortic lesions requires further evaluation.

### 6.4 Others

Besides the agonists mentioned above, many MasR‐related agonists have been successively identified, for example, BAM(8–22), BAM(8–22) TFA, neuropeptide AF (cattle), ML382, MRGPRX1 agonist 1, MRGPRX1 agonist 2, MRGPRX1 agonist 3, MRGPRX1 agonist 4, and MRGPRX4 modulator‐1. However, in practice, studies on these agonists have focused on neurological diseases and autoimmune diseases, and data on cardiovascular and aneurysm‐related diseases are still lacking. Nevertheless, the discovery of these new agonists offers hope for the prevention and treatment of AA.

## 7 FUTURE DIRECTIONS IN THE DEVELOPMENT OF ANG‐(1–7)/MASR‐RELATED DRUGS

The role of the Ang‐(1–7)/MasR axis in the prevention of aortic lesions is exciting. To take full advantage of this discovery, many efforts have been made to improve the efficiency and safety of its usage. For example, during the pre‐clinical (animal) phase of the study, administration was usually by subcutaneous administration via minipumps and later oral delivery was developed.^243^, ^244^, ^245^, ^246^ Similar methods are used for related drugs. However, these traditional usage methods are now considered to be disadvantageous. For example, repeated injections over time may lead to cutaneous calcification as well as subcutaneous tissue fibrosis. In addition, oral administration reduces the effectiveness of the drug and may require higher doses. Drug toxicity in the liver and kidneys from overdoses is still unclear, and there is a lack of studies related to safe dosing. New delivery methods need to be explored for better and safer use of this axis. This section looks at future directions for the Ang‐(1–7)/MasR axis in terms of AA therapy.

### 7.1 Engineered probiotics

Within recent years, another form of Ang‐(1–7) administration has been invented. Carter et al.^247^ developed a genetically engineered probiotic in which a plasmid containing a fragment of the Ang‐(1–7) gene was transferred by electroporation into *Lactobacillus paracasei*, allowing for a continuous effect in the gut after oral administration. This probiotic increased the absolute levels of Ang‐(1–7), ACE and ACE2 and decreased the absolute level of Ang II. Converted to a ratio, it significantly increased the Ang‐(1–7)/Ang II ratio, but did not affect the ACE2/ACE ratio. Multiple subsequent age‐related disease models have confirmed the effectiveness of this delivery method. For example, this engineered probiotic improved many indicators, such as age‐induced motor, cognitive decline, suppressed inflammation, prevented Alzheimer's syndrome, boosted beneficial gut flora, and alleviated diabetic retinopathy.^248^, ^249^, ^250^, ^251^, ^252^ However, some of these improvements were gender‐specific and need to be further investigated.

### 7.2 Targeted therapy

Another issue that needs to be addressed is that of targeting when treating AA. For example, AAA has a specific location of incidence in the abdominal aorta below the diaphragm with 90% concentration in the infrarenal abdominal aorta. Aneurysms can occur in other arterial sites, such as TAAs and brain aneurysms, usually with different pathogenesis. When administered as Ang‐(1–7) or related Mas agonists, the drug may be dispersed throughout the body via the circulatory system, which may reduce efficacy and may also be nontargeted to other organs and regions. If the dosage is elevated, overactivated MasRs may have unpredictable effects. All of this is undesirable when used clinically.

Fortunately, several targeted therapeutic technologies have been developed. For example, exosomes derived from macrophages are often chemotactic.^253^ These exosomes often take on the characteristics of the cells from which they originate and will migrate to regions of injury and inflammation to act. Similarly, some stem cells have homing properties and thus stem cell‐derived exosomes can be used for targeted therapies.^254^, ^255^ It is also possible to make engineered exosomes using genetic techniques, and they often combine the properties of the original cells with additional advantages.^256^, ^257^, ^258^, ^259^ In addition, there are many biomaterials available that also have tissue‐targeting or injury‐targeting properties. When drugs are combined with biomaterials, excellent targeting properties can often be achieved. It not only reduces the dosage of drugs used, but also avoids nontargeting‐induced injuries.

### 7.3 Engineered extracellular vesicles

Extracellular vesicles (EVs) are tiny vesicles with a nanoscale, double‐layered lipid membrane structure secreted outward from the cells, with substrates including nucleic acids, proteins, and metabolites from the source cells.^260^ EVs protect substrates from degradation to a certain extent. For example, exosomes (a form of vesicle) in milk prevent the degradation of substrates miRNA and transport them into the bloodstream of the drinker for further physiological effects.^261^ Ang‐(1–7), as a short‐chain protein, is highly degradable and does not work by oral administration. Similarly, many agonists targeting Mas suffer from this type of problem. EVs offer a solution, although not for all drugs.

## 8 RISKS AND CHALLENGES IN CLINICAL APPLICATION

Multiple studies have confirmed the role of Ang‐(1–7)/MasR in vascular protection but still a way to go before the clinic application, for example, the applicability of AA treatment, safety, and drug delivery modalities. This section will discuss these potential risks and challenges and will suggest possible solutions. It is hoped that it will provide additional references for the development of relevant drugs.

### 8.1 Tumorigenic risk

The Mas gene was initially believed to function as a proto‐oncogene, inducing tumor transformation‐related signaling pathways and driving cell transformation when overexpressed.^262^, ^263^ Although a later study ruled out the suspicion that Mas caused the tumor, which is still doubtful.^264^ Until now, studies have generally concluded that activation of the MasR can alleviate tissue injuries,^265^ but this still does not negate the potential risk of tumorigenesis of the Mas gene. A recent study on male mice found that Ang‐(1–7) directly stimulated the proliferation of bronchial vascular stem cells and alveolar type II cells, indicating a proliferative impact of Ang‐(1–7).^266^ Nevertheless, this study did not establish a connection between MasR activation and cell proliferation. It is unclear whether the beneficial effect of Mas activation on tissue survival is linked with cell proliferation stimulation, which could potentially give rise to unanticipated cellular transformations if excessively activated.

### 8.2 Gender

Like other cardiovascular events, AA is more common in men than in women, likely due to differences in hormone levels and genes.^267^, ^268^, ^269^, ^270^, ^271^ However, there is also a higher incidence of cardiovascular events in men compared with women, and most AA animal models are based on male subjects. Therefore, it is important for future studies to include more female models and to adjust drug dosage and administration methods accordingly.

Recent research based on Drosophila model suggests that the beneficial dose of certain drugs may vary between genders, as seen in a study on a modified Ang‐(1–7) probiotic for Alzheimer's disease where cognitive performance was worsened in female subjects while improving memory deficits in male subjects.^251^ This underscores the need for further exploration into gender‐specific differences in drug efficacy and safety, which is a consensus among researchers not only in AA but also other research fields. Ultimately, it is essential for research to be as balanced and gender‐specific as possible to ensure accurate assessment of drug effectiveness and safety.

### 8.3 Classification

Current research on drugs for AA prevention has focused on animal models, but these models do not fully mimic AA formation in humans. For example, in humans, the location of AAA formation can be classified as suprarenal, subrenal, pararenal, and juxtarenal. The shapes of AAA can be classified as saccular, fusiform, and pseudo. Vessels differ very much from region to region and from shape to shape, and it is not yet known whether the pathology of these AAAs is consistent. Furthermore, the most dangerous forms of AA are vascular rupture and aortic dissection. The differences in the pathological causes of the two conditions have not been determined. At present, it is confirmed that Ang‐(1–7)/MasR certainly reduces vascular injury and prevents AA formation, but the preventive effect on AAs arising from different pathologies may be inconsistent. These deserve more exploration.

## 9 CONCLUSIONS

The formation of AA is associated with varying degrees of injury to the three distinct layers of the arterial wall. Currently, the optimal treatment remains surgical intervention to prevent vessel rupture. Pharmaceutical agents can only mitigate AA‐related risk factors, but there is no robust evidence demonstrating their ability to prevent AA formation. To minimize the incidence of AA and its associated health consequences, regular medical examinations, lifestyle modification, and continued management of cardiovascular disease are recommended. Moreover, the available evidence suggests that many of the pathophysiological mechanisms underlying AAA formation are linked to disorders of the RAS. And activation of the Ang‐(1–7)/MasR axis may effectively mitigate vascular injury and lesions associated with Ang II/AT1R, especially AAA and risk factors associated with AAA, such as endothelial dysfunction and atherosclerosis. Compared with AAA, the lower incidence of other types of AAs results in relatively less research conducted on them, which is also an important direction for future studies. Therefore, Ang‐(1–7)/MasR may be an important target for the prevention and treatment of AA‐related diseases and has an important clinical application potential. Furthermore, existing drug processing and delivery technologies have successfully achieved activation of the Ang‐(1–7)/MasR axis, allowing the challenge of making this axis into an effective therapeutic target to be resolved. Future research could focus on drug delivery modalities and safety assessment. However, it is important to emphasize that the current studies have only demonstrated that the progression of arterial expansion can be slowed down, but that aneurysms that have already formed cannot be reversed. There is still a long way to go to completely treat AA.

## AUTHOR CONTRIBUTIONS

*Writing*: Guang Yang and Abbas Khan *Figures and tables*: Guang Yang *Reviewing and editing*: Wei Liang, Zibo Xiong, and Johannes Stegbauer. All authors have read and approved the final manuscript.

## CONFLICT OF INTEREST STATEMENT

The authors declare that the research was conducted in the absence of any commercial or financial relationships that could be construed as a potential conflict of interest.

## ETHICS STATEMENT

Not applicable.

## Data Availability

Not applicable.
